# Validity of four measures of child care quality in a national sample of centers in Ecuador

**DOI:** 10.1371/journal.pone.0209987

**Published:** 2019-02-14

**Authors:** Florencia Lopez Boo, Marta Dormal, Ann Weber

**Affiliations:** 1 Social Protection and Health Division, Inter-American Development Bank, Washington, District of Columbia, United States of America; 2 Department of Pediatrics, Stanford University School of Medicine, Stanford, California, United States of America; Harvard TH Chan School of Public Health, UNITED STATES

## Abstract

This paper assesses the psychometric properties of four child care quality instruments administered in 404 child care centers in Ecuador: the Classroom Assessment Scoring System for Toddlers, the Infant/Toddler Environment Rating Scale–Revised Edition, the Child Care Infant/Toddler Home Observation for Measurement of the Environment, and the Missouri Infant/Toddler Responsive Caregiving Checklist. We examined their internal consistency, tested the underlying subscale structure by means of confirmatory factor analysis (CFA), verified construct validity by testing associations with quality-related factors (e.g., child-caregiver ratio), and checked concurrent validity of the instruments’ total scores. We found high internal consistency of the instruments at the full scale level and moderate to high at the subscale/domain level. CFA showed high factor loadings, but goodness of fit statistics were low. Construct validity results varied from low to very low depending on the quality-related factor, and concurrent validity from low to very high depending on the instruments compared. This validity exercise provides useful information for policy-makers and researchers interested in using these instruments in the Ecuadorian context or elsewhere in the region. The findings will also inform future research and development of affordable and culturally-appropriate tools for monitoring process quality in child care centers in Latin American countries.

## Introduction

In recent years, countries in Latin America and the Caribbean (LAC) have made a considerable investment in expanding child care coverage for young children. Nonetheless, the quality of child care centers can be quite low [1–3]. Countries in the region face the challenge of ensuring the quality of the child care services they offer, given that children who attend these centers do so during a critical period in their development. In the LAC context, the importance of providing quality early care to children is related to the notion of children’s rights, which is a relatively new concept but one that is widely accepted in the region. The rights approach recognizes children as individuals with legal rights who are equal before any law and policy. The United Nations Convention on the Rights of the Child is the most widely ratified human rights treaty, signed by 194 nations.

Moreover, several experimental and quasi-experimental studies, particularly those conducted in the U.S. and other developed countries, have shown that the impact of child care centers on child development mostly depends on the quality of the centers, especially their process quality. A toolkit by Lopez-Boo and colleagues [4] provides a meta-analysis of this literature. Process variables characterize the quality of child care routines and interactions between children and caregivers, as well as between children and their peers. Process quality is particularly important for children under the age of 3, a critical developmental period in that these children are forming attachments to significant adults [5]. Although young children require less structured curricular content than older children, they demand more individualized attention because they depend more heavily on the caregiver to initiate an interaction until they acquire full mobility [6]. Researchers have found that children in care environments characterized by high process quality are able to initiate and engage in higher-order learning and achieve higher scores on academic achievement tests at a later age as compared to children in low process quality environments [7]. The positive effects of high-quality programs have been found in some cases to last into adulthood [8–13]. Additionally, the literature reveals that indicators of process quality in child care centers are more consistently related to the overall quality of care and children’s developmental outcomes, as compared to structural indicators such as group size, caregiver demographics, center safety, and materials [10,14–16].

The same process variables that are vital to ensuring quality care for infants and toddlers represent one of the LAC region’s greatest shortcomings [2] and are also the most difficult variables to measure. These variables require expert observation, judgment and interpretation, which is complex, time-consuming and costly. This partly explains why monitoring strategies for child care services in the LAC region use checklist measures that primarily focus on structural quality instead of process quality. The former are easily quantifiable aspects of care such as the number of books in the classroom, whereas the latter are less concrete indicators such as caregivers’ responsiveness and children’s engagement in play. This approach is in contrast to the Quality Rating and Improvement System (QRIS) in the U.S., a systemic approach to assess, improve, and communicate the level of quality in early and school-age care and education programs. QRIS award quality ratings to early and school-age care and education programs that meet a set of defined program standards. Moreover, empirical evidence shows that direct observation instruments used to evaluate service quality predict child outcomes to a significantly greater degree than interviews or checklists [17].

To fill a void in the LAC literature, Araujo and colleagues [1] reported on different aspects of service quality at *Centros Infantiles del Buen Vivir* (CIBVs) in Ecuador. The CIBVs were the primary providers of public child care services in Ecuador for infants and toddlers at the time of the study. According to CIBV administrative data, in 2011 these centers served some 140,000 children in approximately 3,800 centers throughout the country [18]. The service mainly operates under third-party agreements with local governments, community organizations, foundations, churches, etc., who receive a transfer of public resources to cover operating costs. Some of these entities supplement public funds with their own resources. Unlike most child care services in the region, at the time of data collection this program functioned in both rural and urban areas. Caregivers are hired by the sub-contracting organization and earn minimal wages.

The Ecuadorian centers face two major challenges for delivering high quality services. The first relates to the classroom composition: centers group children into classrooms with a very broad range of ages from 6 months to 3 years, extending up to 5 years with children who are eligible for mandatory preschool [1]. In a setting where the staff members are typically not professionals, this mix of ages complicates the task of child care. The second challenge relates to staff training in terms of their knowledge and specific relevant skills. Although caregivers are officially required to have completed secondary school, in practice, compliance with this requirement is lax.

In their study in 2012, Araujo and colleagues [1] administered a battery of widely-used instruments to measure the quality of child care services in the CIBVs, including four internationally-recognized quality instruments that were then adapted for use in Ecuador. The four selected were the instruments most commonly used in the preceding two decades in U.S. child care centers and elsewhere. The instruments ranged in complexity and quality focus and included the Classroom Assessment Scoring System for Toddlers (CLASS) [19], the Infant/Toddler Environment Rating Scale–Revised Edition (ITERS-R) [20], the Child Care Infant/Toddler Home Observation for Measurement of the Environment (CC-IT-HOME) [21], and the Missouri Infant/Toddler Responsive Caregiving Checklist (MITRCC) [22]. They all have comparable domains of quality and some of them, such as CLASS and ITERS-S have been proven to be strongly predictive of child development (See Lopez Boo and colleagues [4] for a review of the evidence). It is worth noting that CLASS and ITERS-R have been designed for center-based care only, while CC-IT-HOME and MITRCC could be used (in different versions of the instrument) both in center-based and home-based early childhood programs.

The primary objective of this study was to assess the psychometric properties of these four instruments in their first application in Ecuador. For each instrument, we examined internal consistency, tested the underlying subscale structure by means of confirmatory factor analysis (CFA), and, due to the lack of child development indicators as validators in our data, we verified construct validity by testing associations with socio-economic status and structural quality-related factors. We also checked concurrent validity by comparing each instrument total score with the total scores of the other instruments on the same CIBV sample. Apart from a study by Kane and Staiger [23] for the U.S., we found no other studies that have undertaken this type of exercise, in LAC or elsewhere, because of the difficulty of administering such a diverse set of measures to a single sample.

## Materials and methods

### Participants

The sampling frame consisted of all child care centers in the administrative databases of Ecuador’s CIBV in May 2012. That year, these databases contained information on 3,575 centers, including data on the number of children enrolled and the number of community staff that worked at these centers. The sample was stratified into two groups of centers according to their child-caregiver ratio given that the literature identifies this ratio as a key structural quality variable. Specifically, we divided centers with ratios above and below the sample median ratio of 9.2 children per adult (1,779 and 1,776 centers, respectively). About 202 centers were randomly chosen from each stratum, for a final sample of 404 centers.

### Measures

The four instruments assessed in this study are characterized as providing a holistic measurement of child care quality but differ by domain of quality captured and relative complexity. Table 1 shows the general features of the instruments. We briefly discuss some of them. A more detailed description of these four instruments and their characteristics, costs, advantages and disadvantages is presented in the toolkit by Lopez-Boo and colleagues [4].

**Table 1:** Main characteristics of quality instruments.

	CLASS	ITERS-R	CC-IT-HOME	MITRCC
Age range (months)	15–36	0–30	0–36	0–36
Number of domains	2	N/A	N/A	N/A
Number of dimensions/scales	8 dimensions	7 scales	6 scales	N/A
Number of items	N/A	39	43	20
Type of variables	Process	Structural/process	Structural/process	Structural/process
Assessment method	Observation (direct or video)	Direct observation and reporting	Direct observation and reporting	Direct observation
Official training	Yes	No	No	No
Scoring method	1–7	1–7^a^	0–1 (yes/no)	0–1 (yes/no)
Minimum administration time (hours)	2^b^	3.5	1	3.5
Total cost (in US$)^c^	902.90	22.90	40.30	0.00

^a^ITERS-R scoring is administered gradually, meaning that once a classroom fails to comply with a subset of items within a given subscale, it receives the score corresponding to the highest stop point.

^b^Unlike other instruments, administration begins with classroom video footage of a four-hour day, from which four 20-minute observation cycles are extracted. In addition, CLASS is the only instrument that is not scored in the field and that requires a post-fieldwork phase for the coding of videos.

^c^This cost corresponds to the cost of materials per observer/coder. The only exception is CLASS, for which the cost of the mandatory, official train-the-trainer program is included. Prices were valid as of December 28, 2015. Further research and prior evidence on psychometric properties in other settings and references to other countries where the tools were implemented can be found in Tables 3 (ITERS-R), 4 (CLASS), 8 (MITRCC) and 9 (CC-IT-HOME) in Lopez-Boo and colleagues [4].

#### Domains of quality

The CLASS explores eight dimensions in two domains of quality: Emotional and Behavioral Support (dimensions 1 to 5), and Facilitation of Learning and Development (dimensions 6 to 8). The ITERS-R and the CC-IT-HOME are organized into seven and six subscales of quality, respectively. The MITRCC consists of 20 items that are not organized into subscales or dimensions of quality. CLASS is the only one out of the four instruments that focuses exclusively on process variables (child-caregiver interactions, specifically).

#### Administration method

Both the CLASS (by filming and coding off-site) and the MITRCC (by coding on-site in the center) collect data exclusively through observation, while the other two instruments combine observation with information reported by the caregiver in the classroom under study. Observation demands more time, training and resources than caregiver-report, but generally produces higher-quality data than reported information [4]. Filming and coding off-site has the advantage that the video coder can focus on what is happening with the child, blocking out what is going on around her (e.g., other groups of children who are not part of the sample or the activities of support staff). However, this method might be more costly due to the required camera equipment.

#### Assessor training

Out of the four instruments, only CLASS requires formal training by the publisher. Post-secondary education is also usually required for observers in all the instrument manuals.

#### Scoring

The complexity of the scoring method varies widely across the four instruments. For CLASS, each dimension is assigned a score ranging from 1 to 7. The ITERS-R also has a 1 to 7 scoring system designed to be administered gradually, and with complex stopping rules. In contrast, the CC-IT-HOME and MITRCC use a binary (yes/no) response for each of a set of items. A CLASS score of 1 or 2 indicates low quality; 3 to 5, medium quality; and 6 or 7, high quality. For the ITERS-R subscales and total scale, inadequate quality is indicated by a score of 1; minimal quality by a score of 3; good quality is scored 5; and excellent quality is scored 7. For the MITRCC, a total score of 6 or below indicates minimal quality; 7, average quality; 8, above-average quality; and 9 or more, high quality. The CC-IT-HOME manual, on the other hand, does not define the center’s level of quality based on the score obtained.

Related to the complexity of the scoring method is the depth, or richness, with which instruments seek to evaluate the various caregiver-child interaction constructs. In the case of the Listening and Talking subscale of the ITERS-R and the Caregiver Responsivity subscale of the CC-IT-HOME, for example, while both aim to capture the quality of child-directed speech by caregivers, the former requires the interviewer to evaluate indicators that are more abstract or difficult to observe and tease out (e.g., “Staff use a wide range of simple exact words in communicating with children”). The latter, on the other hand, only requires the interviewer to tally the number of actions (e.g., “Caregiver spontaneously speaks/vocalizes to child at least twice”). CLASS dimensions are also complex to evaluate.

#### Administration time

The complexity of the instrument is also reflected in the amount of time required for administration. The CC-IT-HOME, for example, can be administered in the field in just one hour, while a minimum of two hours of video-recording in the centers is recommended for CLASS to produce reliable estimates. While a total of three and a half hours is required for both the ITERS-R and the MITRCC, the latter also requires greater competence of the observer in correctly identifying certain complex constructs, such as object permanence, underlying specific activities or interactions that may take a long time to occur in a classroom.

#### Administration cost

CLASS has the highest administration costs, mainly due to the mandatory official training. However, the time spent by observers to achieve inter- and intra-rater reliability is not considered here for the costing of any of the instruments. Given that a single trained coder of the CLASS can code many classrooms, the CLASS has the potential for large economies of scale.

### Procedures

Because our quality measures were administered in a context different from the one for which they were originally designed and validated, we undertook an adaptation process to ensure their cultural and linguistic equivalence. We translated the original version of all instruments into Ecuadorian Spanish, then using a second translator, translated the text back into the source language. The Spanish version was considered accurate when comparing the original version to the back-translation. A bilingual, bicultural native speaker from Ecuador was involved throughout the entire process. Most items were deemed culturally appropriate by the team of local researchers who knew the context well, and only minor modifications were made. For example, one item in the Acceptance scale of the CC-IT-HOME and six items from MITRCC were removed because they were not applicable to the CIBVs context. Another item from the MITRCC was disaggregated to facilitate its administration.

During the fieldwork phase, each center was visited for a full day by a pair of researchers responsible for collecting data on one group of children and their caregiver and for administering the quality measures. The group was composed of children who were under the age of 36 months at the beginning of the school year. If the center only had one group of children in this age range, then that group constituted the study group. If there was more than one group that included children under the age of 36 months, priority was given to the one in which all of the children fell within that range. If there were either multiple groups in which all of the children were under 36 months or no group in which all of the children were under 36 months, one group was selected at random. Once the selection was made, the researchers focused on the selected group for the rest of the day.

All of the measures were administered in the same order and at the same time of day to ensure comparability across centers (the CLASS, ITERS and MITRCC between 8am and 12pm; and the HOME at 12pm). For each pair of interviewers that visited the CIBVs, one was responsible for filming the group, administering the MITRCC via direct observation and conducting interviews, and the other for administering the CC-IT-HOME and ITERS-R, both via direct observation. The administration of the CLASS was performed using classroom video footage of a four-hour day, from which four 20-minute segments were extracted following the CLASS editing protocol. The field research team was solely responsible for shooting the videos that were subsequently evaluated by a team of seven certified CLASS coders. Each video segment was coded twice by two different people who were randomly assigned to the task. A segment was coded a third time if the difference between the first two coders was more than two points for dimensions with less variability (Negative Climate, Regard for Child Perspectives, Quality of Feedback, and Language Modeling) or three points for dimensions with more variability (Positive Climate, Teacher Sensitivity, Behavior Guidance, Facilitation of Learning and Development). For scores requiring a third coder, the two ratings with the smallest discrepancy between them were retained and the ratings of the most divergent coder were dropped.

The assessors who administered the MITRCC were experienced interviewers with completed secondary education. Those responsible for administering the CC-IT-HOME, the ITERS-R, and the CLASS coders were researchers with post-secondary education in the field of child psychology or early childhood education. The training was organized into two days of theory training: one day and a half for the ITERS-R and half a day for the CC-IT-HOME. Reliability was evaluated over eight days, with pairs of individuals going out to the field with the trainer (i.e., the gold standard). After the pilot, from a total of 14 potential assessors, five reliable enumerators were selected to administer ITERS-R and CC-IT-HOME. For the CLASS, coders completed eight days of training and practice, followed by seven days during which they worked on group coding exercises that enabled them to calibrate their coding. In addition, the coders were accompanied throughout the entire coding process by a certified CLASS trainer, with whom they performed daily group coding practice as part of a continuous training process and to ensure that Inter-Rater Reliability (IRR) was maintained. IRR was measured during the training and pilot testing of the instruments as the percentage agreement between both coders at the item level. CLASS IRR was 90% for all videos and all coders. CC-IT-HOME’s IRR was 88% for all assessors and ITERS-R 86% for all assessors. The lowest IRR was for MITRCC at 56% on average, most likely because, as previously described, its items are abstract and difficult to observe and tease out. For more details on sample selection and the fieldwork phase, see Araujo and colleagues [1].

### Analytical strategy

We tested the internal consistency of the instruments using Cronbach’s alpha (*α*) and Pearson correlation coefficients (*r*) between domain/scale score and total score.

We conducted CFA using a robust ML estimator (MLR) for the CLASS, ITERS-R and CC-IT-HOME because the data were non-normal and continuous, and the weighted least squares, mean- and variance-adjusted (WLSMV) estimator for the MITRCC because the data were non-normal and binary. We assessed the fit of the measurement models using the Chi-Square, the Root Mean Square Error of Approximation (RMSEA), the Comparative Fit Index (CFI), the Tucker-Lewis Index (TLI), and the Standardized or Weighted Root Mean Square Residual (SRMR and WRMSR, respectively). We considered the following cut-offs for the goodness of fit: p-value>0.05 for the Chi-Square, RMSEA <0.08, CFI and TLI ≥0.90, and SRMR <0.08.

In the absence of child development outcomes in our data, we tested construct validity using multiple factors expected to be related with process quality, including proxies for family socioeconomic status. Previous research has shown that the poorest children generally receive lower-quality child care services [3]. However, as no household questionnaire was administered to the families of the children in the child care centers, the available socioeconomic indicators were limited to geographical location of the center (urban or rural), proportion of indigenous children in each group, and whether, on average, families paid a subscription fee equal to or higher than $5 to the center. The latter indicator was used because evidence shows that in public (i.e., free) childcare services in LAC, about a third of them expect operators to supplement the public funding they receive with parental contributions [18]. This is also the case for the CIBVs where contributions are voluntary, and in some cases an arbitrary fee is set by the center based on the socioeconomic status of families they serve. We also used various proxies for structural quality. Structural variables have been shown in the literature to be significantly correlated to child care quality [6], particularly that higher child-caregiver ratios tend to be associated with a lower frequency of positive affective interactions [24]. We therefore constructed an indicator of whether the center had a child-caregiver ratio below the sample median (*M* = 9.7). Studies have also shown that caregivers with more years of education and training in early childhood education are more likely to use activities that are more stimulating and are better-suited to the development of the children in their care [25, 26]. We used the following caregiver characteristics: her age, her years of education, years of experience in that CIBV, whether she received training by the center, whether she is indigenous, and her score on three instruments designed to measure her knowledge of child development and caregiver practices: the Teacher Practice Survey [27], the Knowledge of Infant Development Inventory (KIDI) [28], and the Infant-Toddler and Family Instrument (ITFI) [29]. For continuous indicators, we used Pearson correlations to assess their relationship with instrument scores. For dichotomous variables, two-tailed *t*-tests were performed to test for differences between the mean scores of the two groups (e.g., centers located in urban vs. rural areas). Standard errors of the means were corrected for clustering at the canton level (equivalent of a township).

Finally, Pearson correlations were used for concurrent validity between the instruments’ total scores.

We consider CFA loadings (*λ*) and correlation coefficients to be very high when in the range of 0.80 to 1; high in the range of 0.60 to 0.80; moderate in the range of 0.40 to 0.60; low in the range of 0.20 to 0.40; and very low when less than 0.20.

The CFA were estimated in MPlus (version 7). The rest of the analyses were conducted in Stata (version 14).

## Results

### Descriptive statistics

#### CIBV characteristics

Table 2 presents the characteristics of all the centers in our sample. Araujo and colleagues [1] compared the sampled population of centers to those of the full population from which they were drawn.

**Table 2:** Sample characteristics.

	M/Proportion	SD
Child-caregiver ratio	9.46	1.80
Total number of children	31.18	18.29
0–12 months	2.41	2.37
13–24 months	7.08	4.73
25–36 months	9.02	6.35
37 months and older	12.66	10.41
Total number of staff	5.69	2.72
Caregivers	3.25	1.64
Food service staff	1.38	0.75
Center is in urban area (%)	0.46	0.50
Has own electricity meter (%)	0.63	0.48
Has drinking water (%)	0.56	0.50
Has a sewerage system (%)	0.59	0.49
Type of operating entity^a^ (%)		
Municipality	0.32	0.47
Foundation/NGO/committee/religious ent.	0.24	0.42
Parish council	0.24	0.43
Central government	0.02	0.14
INFA	0.13	0.34
Other	0.18	0.38

M = mean, SD = standard deviation

^a^46 centers report >1 operating entity

Child-caregiver ratios were high (approximately 9 children per caregiver). As mentioned before, centers provided care to a broad age range, including children above 36 months who are eligible for mandatory preschool. Access to public services was low: 63% had their own electricity meter, 56% drinking water, and 59% had a sewerage system. Close to half of the centers were in urban areas. Centers were managed under third-party agreements by various entities, with the three most common categories being municipalities, foundations or NGOs, and parish councils.

#### Quality scores

Table 3 summarizes the mean scores of the instruments across the 404 centers, as well as the scores at the 10^th^ and 90^th^ percentiles.

**Table 3:** Instrument scores for child care centers in Ecuador.

Instrument	M	SD	Possible range	P10	P90
CLASS					
1. Positive climate	3.34	0.62	[1–7]	2.63	4.13
2. Negative climate	6.61	0.44	[1–7]	6.00	7.00
3. Teacher sensitivity	3.36	0.63	[1–7]	2.63	4.13
4. Regard for child perspectives	1.98	0.28	[1–7]	1.67	2.38
5. Behavior guidance	2.85	0.50	[1–7]	2.25	3.50
6. Facilitation of learning and development	2.08	0.53	[1–7]	1.50	2.75
7. Quality of feedback	1.30	0.33	[1–7]	1.00	1.75
8. Language modeling	1.56	0.51	[1–7]	1.00	2.25
Total score	2.88	0.42	[1–7]	2.44	3.35
ITERS-R	** **	** **	^** **^	** **	** **
1. Space and furnishings	2.10	0.62	[1–7]	1.40	3.00
2. Personal care routines	1.69	0.54	[1–7]	1.00	2.50
3. Listening and talking	2.48	1.18	[1–7]	1.00	4.50
4. Activities	1.54	0.47	[1–7]	1.00	2.22
5. Interaction	3.30	1.26	[1–7]	1.50	5.00
6. Program structure	2.57	1.26	[1–7]	1.00	4.33
7. Parents and staff	2.00	0.64	[1–7]	1.17	2.83
Total score	2.08	0.53	[1–7]	1.41	2.85
CC-IT-HOME	** **	** **	^** **^		
1. Caregiver responsivity	6.68	2.52	[0–11]	3.00	10.00
2. Acceptance	5.69	0.59	[0–6]	5.00	6.00
3. Organization	2.85	0.99	[0–6]	2.00	4.00
4. Learning materials	4.47	2.09	[0–9]	2.00	7.00
5. Caregiver involvement	3.63	1.76	[0–9]	1.00	6.00
6. Variety of stimulation	1.37	0.79	[0–4]	1.00	3.00
Total score	24.69	6.06	[0–42]	17.00	33.00
MITRCC	4.52	2.23	[0–10]	1.50	7.50

M = mean, SD = standard deviation, P10 = 10th percentile, P90 = 90th percentile

For the most part, centers provide low-quality child care. This low quality was particularly observed with the CLASS, whose scores at the aggregate level fell within the range of low to medium quality (*M* = 2.88, *SD* = 0.42) and were concentrated almost exclusively in the bottom two-thirds of the distribution, and with the ITERS-R, whose total score did not reach the minimal quality range (*M* = 2.08, *SD* = 0.53). These results were also observed at the subscale/dimension level of the two measures. For CLASS, five of the eight dimensions had scores indicating low quality, although most of the centers fell within the medium quality range for the dimensions grouped under the Emotional and Behavioral Support domain. On the ITERS-R, six of the seven subscales failed to reach a minimal level of quality.

Even when considering only the best CIBVs—those that fall in the 90^th^ percentile of the distribution for the ITERS-R total score—a minimal quality level was barely attained (*M* = 2.85). In addition, for both the CLASS and ITERS-R, the scores showed very little dispersion. Nevertheless, there were two favorable exceptions: (a) the Negative Climate dimension of the CLASS (reverse coded) reflected a very low to nonexistent level of expressed negativity in the centers; and (b) the best centers in the sample (those in the 90^th^ percentile of the distribution) achieved a score of 5 on the Interaction subscale of the ITERS-R, a level of quality considered to be good. For the MITRCC, centers were on average at the minimal quality level, while those at the 90^th^ percentile had an average quality level. The average scores on the subscales of the CC-IT-HOME, while also reflecting a generally low level of quality, were more scattered than the scores of the other instruments.

### Psychometric properties

To assess the psychometric properties of the four instruments in their first application in Ecuador, we present evidence for internal consistency, goodness of fit to the publisher’s measurement model using CFA, construct validity, and concurrent validity.

#### Internal consistency

Table 4 shows Cronbach’s alphas at the scale and total level, and Pearson correlations between each scale and the total score.

**Table 4:** Internal consistency of quality instruments.

Instrument	Alpha	Correlation
CLASS		
Emotional and Behavioral Support	0.86	0.97***
Engaged Support for Learning	0.90	0.91***
Overall	0.91	
ITERS-R^a^		
1. Space and furnishings	0.61	0.64***
2. Personal care routines	0.49	0.69***
3. Listening and talking	0.61	0.76***
4. Activities	0.63	0.66***
5. Interaction	0.77	0.82***
6. Program structure	0.76	0.78***
7. Parents and staff	0.57	0.65***
Overall	0.85	
CC-IT-HOME		
1. Caregiver responsivity	0.74	0.83***
2. Acceptance	0.51	0.32***
3. Organization	0.26	0.53***
4. Learning materials	0.66	0.74***
5. Caregiver involvement	0.66	0.74***
6. Variety of stimulation	0.35	0.53***
Overall	0.83	
MITRCC	0.85	

Pearson correlation coefficients between scale/domain and total score.

*** p<0.10.

^a^Cronbach alpha’s excludes items 23, 32 and 36 that only applied to 57, 19 and 44 centers, respectively.

Overall, we observed high internal consistency of the instruments for the full scale: all *α* > 0.8. Results were moderate to high at the scale/domain level (with two exceptions for the CC-IT-HOME in the low range), in the range of *α* = [0.86–0.91] for the CLASS, *α* = [0.49–0.77] for the ITERS-R and *α* = [0.26–0.74] for the CC-IT-HOME. The correlation coefficients between each of the subscales with the total score for the same instrument were on average *r* = 0.94 for CLASS, *r* = 0.71 for the ITERS-R and *r* = 0.62 for the CC-IT-HOME.

#### Fit to published measurement model

**Fig 1:**
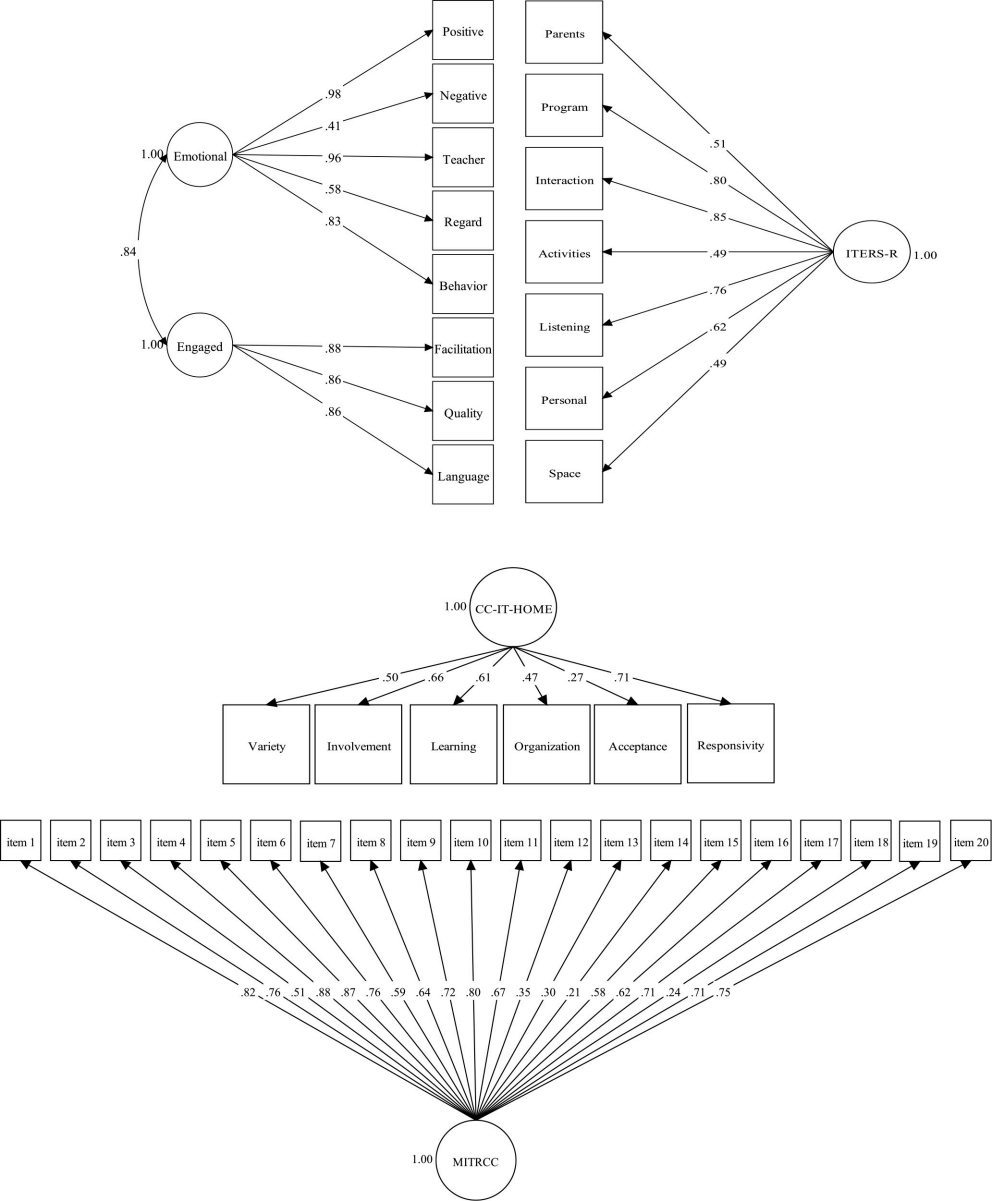
Confirmatory factor analysis for publishers' framework. Factor loadings, standardized estimates. Robust ML estimator (MLR) for the CLASS, ITERS-R and CC-IT-HOME, and WLSMV estimator for the MITRCC.

Measurement models were defined *a priori* based on the hypothesized structure set forth by the instrument developers and depicted as separate path diagrams in Fig 1. A single latent construct model is used for testing the ITERS-R and CC-IT-HOME, in keeping with the developers’ theoretical structure for these measures [20, 21]. The CLASS, on the other hand, was tested with two correlated latent domains for quality, Emotional and Behavioral Support and Engaged Support for Learning, as described previously [19]. As the publishers of the MITRCC do not organize the instrument into subscales, we mapped all items to a single latent construct [22].

Factor loadings were all statistically significant at the 1% level. For CLASS, factor loadings for the three subscales on Engaged Support for Learning and for three of the five subscales on Emotional and Behavioral Support were very high (*λ* = [0.83–0.98]). The loading for Regard for Child Perspectives was borderline high (*λ* = 0.58), but only moderate for Negative Climate (*λ* = 0.41). The standardized covariance between the two latent constructs was high at 0.84. For ITERS-R, factor loadings for three of the subscales, specifically Listening and Talking, Interaction, and Program Structure, were high to very high (*λ* = [0.76–0.85]), while the other four were moderate to high (*λ* = [0.49–0.62]). Factor loadings for the CC-IT-HOME were moderate to high (*λ* = [0.47–0.71]) except for the Acceptance subscale which was low (*λ* = 0.27). As with Negative Climate and CLASS, the Acceptance subscale in the CC-IT-HOME demonstrated very little variation with 74% of the sample achieving a perfect score. The MITRCC had four very high loadings (*λ* = [0.80–0.88]), nine in the high range (*λ* = [0.62–0.76]), and three in the moderate (*λ* = [0.50–0.59]) and low ranges (*λ* = [0.21–0.35]).

Although factor loadings were mostly high, model fit was usually poor. All Chi-Square statistics had a p-value = 0.00. RMSEA was below the cut-off only for the MITRCC, while they were higher for the CLASS at 0.10, 0.12 for ITERS-R and 0.09 for the CC-IT-HOME. CFI was above the cutoff for all models, but results were borderline (between 0.91 and 0.95). Results were also around the cut-off for the TLI (between 0.86 and 0.93). All SRMR were below the cut-off of 0.08.

#### Construct validity

Table 5 shows Pearson correlation coefficients between instrument scores and center and caregiver characteristics.

**Table 5:** Correlations between instruments, sociodemographic indicators and proxies of structural quality.

	% indigenous children	Age (years)	Education (years)	Time working in center (years)	Salary	Teacher practice survey (z-score)^a^	KIDI (z-score)^b^	IFTI (z-score)^c^
CLASS	** **							
1. Positive climate	-0.19***	0.01	0.15***	-0.02	0.08*	0.28***	0.14***	0.13**
2. Negative climate	0.02	-0.01	0.05	-0.02	0.02	0.17***	-0.04	0.13***
3. Teacher sensitivity	-0.20***	0.01	0.16***	-0.01	0.12**	0.25***	0.12**	0.14***
4. Regard for child perspectives	-0.04	-0.05	-0.02	-0.04	0.01	0.20***	0.15***	0.06
5. Behavior guidance	-0.19***	0.02	0.25***	0.03	0.05	0.32***	0.06	0.16***
6. Facilitation of learning and development	-0.15***	0.04	0.19***	0.07	0.13***	0.34***	0.05	0.13**
7. Quality of feedback	-0.16***	0.05	0.16***	0.08	0.08	0.29***	0.02	0.10**
8. Language modeling	-0.23***	0.11**	0.18***	0.07	0.06	0.29***	0.04	0.10**
Total score	-0.16***	0.05	0.15***	0.04	0.09*	0.33***	0.10*	0.17***
ITERS-R								
1. Space and furnishings	-0.15***	0.01	0.12**	0.02	0.01	0.13***	0.06	0.28***
2. Personal care routines	-0.17***	0.06	0.17***	0.06	0.08	0.21***	0.12**	0.22***
3. Listening and talking	-0.08	0.05	0.10*	0.01	0.04	0.17***	0.09*	0.28***
4. Activities	-0.11**	-0.08	0.15***	-0.01	0.10*	0.24***	0.05	0.14***
5. Interaction	-0.08	0.10**	0.18***	0.06	0.11**	0.13***	0.13***	0.35***
6. Program structure	-0.09	0.10**	0.13**	0.10**	0.07	0.02	0.13***	0.39***
7. Parents and staff	-0.08*	0.02	0.06	0.00	0.03	0.03	0.05	0.16***
Total score	-0.13***	0.06	0.18***	0.04	0.09*	0.18***	0.13**	0.36***
CC-IT-HOME								
1. Caregiver responsivity	-0.18***	0.12**	0.15***	0.04	0.11**	0.07	0.13***	0.33***
2. Acceptance	0.02	0.06	0.00	-0.06	0.10**	0.02	0.07	0.05
3. Organization	-0.07	0.05	0.21***	0.07	0.18***	0.08	0.14***	0.23***
4. Learning materials	-0.04	0.04	0.14***	0.07	0.07	0.19***	0.09*	0.35***
5. Caregiver involvement	-0.09*	0.06	0.15***	0.04	0.10**	0.06	0.17***	0.21***
6. Variety of stimulation	-0.14***	0.08	0.13***	-0.05	0.08*	0.28***	0.19***	0.08*
Total score	-0.14***	0.10**	0.21***	0.05	0.15***	0.16***	0.19***	0.37***
MITRCC	-0.12**	0.11**	0.15***	0.07	0.11**	0.15***	0.12**	0.39***

Pearson correlation coefficients.

* p<0.01

** p<0.05

*** p<0.10.

^a^Teacher Practice Survey [27]

^b^Knowledge of Infant Development Inventory [28]

^c^Infant-Toddler and Family Instrument [29]

Overall, correlations were of the expected sign and were significant except for the age of the caregiver and the time she had been working in the center, both of which showed few significant correlations with the subscale scores. Magnitudes were however mostly very low or low, with only a few reaching the moderate range. Results were highest for the Teacher Practice Survey with the CLASS, and the IFTI with the ITERS-R and CC-IT-HOME approaching magnitudes of *r* = 0.4.

In Table 6 we present mean instrument scores by center and caregiver characteristics and a p-value for the difference between the groups.

**Table 6:** Instrument scores by sociodemographic indicators and proxies of structural quality.

Instrument	Child-caregiver ratio < median			Center is urban			Family pays ≥$5			Caregiver received training			Caregiver is indgenous		
	Yes	No		Yes	No		Yes	No		Yes	No		Yes	No	
CLASS	M	M	Pval	M	M	Pval	M	M	Pval	M	M	Pval	M	M	Pval
1. Positive climate	3.40	3.28	0.09	3.44	3.26	0.01	3.63	3.27	0.00	3.37	3.25	0.06	3.16	3.44	0.00
2. Negative climate	6.66	6.57	0.02	6.60	6.63	0.57	6.60	6.62	0.79	6.63	6.57	0.20	6.58	6.63	0.41
3. Teacher sensitivity	3.42	3.31	0.11	3.45	3.29	0.03	3.65	3.30	0.00	3.40	3.26	0.05	3.17	3.47	0.00
4. Regard for child perspectives	2.00	1.97	0.23	2.00	1.97	0.57	2.01	1.98	0.47	2.00	1.95	0.09	1.95	2.00	0.14
5. Behavior guidance	2.89	2.82	0.26	2.93	2.79	0.04	3.05	2.81	0.00	2.89	2.76	0.03	2.70	2.94	0.00
6. Facilitation of learning and development	2.14	2.02	0.03	2.14	2.03	0.09	2.18	2.06	0.10	2.10	2.03	0.35	1.97	2.14	0.00
7. Quality of feedback	1.32	1.29	0.47	1.34	1.27	0.09	1.37	1.29	0.07	1.32	1.25	0.03	1.23	1.34	0.00
8. Language modeling	1.58	1.54	0.43	1.64	1.50	0.01	1.67	1.54	0.11	1.59	1.50	0.18	1.40	1.65	0.00
Total score	2.92	2.83	0.03	2.92	2.84	0.11	2.98	2.86	0.03	2.91	2.79	0.01	2.77	2.94	0.00
ITERS-R															
1. Space and furnishings	2.10	2.11	0.81	2.22	2.01	0.00	2.27	2.07	0.23	2.13	2.02	0.07	1.99	2.17	0.02
2. Personal care routines	1.71	1.67	0.44	1.81	1.59	0.00	1.83	1.66	0.01	1.74	1.54	0.00	1.56	1.76	0.00
3. Listening and talking	2.51	2.45	0.63	2.59	2.39	0.12	2.76	2.42	0.05	2.60	2.15	0.00	2.31	2.58	0.09
4. Activities	1.58	1.50	0.11	1.59	1.50	0.12	1.69	1.50	0.06	1.56	1.48	0.09	1.49	1.57	0.13
5. Interaction	3.35	3.24	0.42	3.53	3.10	0.00	3.55	3.25	0.07	3.49	2.74	0.00	3.17	3.37	0.18
6. Program structure	2.62	2.51	0.41	2.78	2.39	0.01	2.78	2.53	0.12	2.75	2.04	0.00	2.62	2.54	0.60
7. Parents and staff	2.01	2.00	0.91	2.11	1.91	0.00	2.22	1.96	0.07	2.04	1.89	0.03	1.95	2.03	0.27
Total score	2.11	2.06	0.34	2.20	1.99	0.00	2.27	2.04	0.03	2.15	1.88	0.00	2.00	2.13	0.04
CC-IT-HOME															
1. Caregiver responsivity	6.67	6.69	0.94	7.18	6.26	0.00	6.99	6.61	0.23	6.83	6.24	0.03	6.01	7.05	0.00
2. Acceptance	5.66	5.72	0.42	5.70	5.68	0.70	5.72	5.68	0.54	5.72	5.60	0.09	5.71	5.68	0.70
3. Organization	2.81	2.90	0.35	2.99	2.74	0.01	3.12	2.79	0.01	2.95	2.59	0.00	2.79	2.89	0.46
4. Learning materials	4.55	4.40	0.49	4.97	4.05	0.00	4.96	4.36	0.08	4.65	3.96	0.00	4.29	4.57	0.25
5. Caregiver involvement	3.87	3.38	0.01	3.92	3.36	0.01	3.95	3.54	0.09	3.70	3.38	0.05	3.48	3.70	0.33
6. Variety of stimulation	1.44	1.30	0.12	1.48	1.28	0.01	1.42	1.36	0.50	1.43	1.20	0.00	1.20	1.47	0.00
Total score	25.00	24.38	0.34	26.24	23.37	0.00	26.15	24.35	0.01	25.27	22.98	0.00	23.48	25.35	0.00
MITRCC	4.56	4.47	0.71	5.06	4.06	0.00	4.86	4.44	0.15	4.73	3.90	0.00	4.02	4.79	0.00

M = mean, Pval = p-value of *t*-test for difference in means. Clustering at canton (township) level.

Results showed higher scores for centers with child-caregiver ratios below the median, in urban areas, where families pay at least $5 for subscription and where the caregiver received training, and lower scores where the caregiver was indigenous, with very few exceptions. However, these differences were not always statistically significant. Centers with child-caregiver ratios below the median had significantly higher scores only for some of the CLASS dimensions but not for the other instruments. Results for centers where families pay a $5 fee or more and where the caregiver was indigenous were mixed across instruments, although for the latter they were particularly lower and significant for the CLASS. On the other hand, scores were higher and the differences were significant across most dimensions/scales for centers in urban areas and where the caregiver received on-the-job training, particularly for the ITERS-R, CC-IT-HOME and MITRCC.

#### Concurrent validity

Correlations between the overall CLASS score and the other three measures were low but statistically significant: *r =* 0.34 with ITERS-R and MITRCC, and *r* = 0.36 with CC-IT-HOME. The correlation between the ITERS-R and CC-IT-HOME, on the other hand, was very high (*r* = 0.80). This was expected given that they both measure some structural quality indicators even though their focus is on process quality, and that they both combine observation and survey methods. Furthermore, their field observer profile is identical. The correlations of the MITRCC with the ITERS-R and CC-IT-HOME were moderate (*r* = 0.54 and *r* = 0.57, respectively).

## Discussion

This unique study analyzed data obtained from four instruments that are widely used to measure child care quality for children ages 0 to 36 months, from a nationally representative sample of 404 child care centers in Ecuador. To our knowledge, an analysis of such a varied set of quality measures—administered in the same classrooms, at the same time of day—has never before been performed. The operational design of the fieldwork was unique and fills an important void in the literature, particularly in LAC.

Overall process quality in the Ecuadorian centers was poor. The total CLASS scores were medium quality or below, and the ITERS-R total scores failed to reach minimal quality. A recent evaluation of the U.S. Early Head Start centers, which serve children under the age of 3 from low-income families, found that these U.S. centers fell in the moderate to high-quality range on the Toddler CLASS instrument [30]. This difference across countries is not surprising considering the Ecuadorian context differs from the U.S. context not only by age and sociodemographic composition of classrooms, but also by how (weakly) protocols are implemented.

Scales and dimensions generally loaded strongly onto latent constructs. Two exceptions were Negative Climate for CLASS and Acceptance for the CC-IT-HOME, which was consistent with our fieldwork observations that the overall level of expressed negativity in the Ecuadorian classrooms was indeed very low (i.e., there was insufficient variability along these dimensions to exploit). The goodness of fit statistics on the other hand were poor, suggesting that alternative frameworks to those proposed by the publishers could be explored for Ecuador.

For construct validity, correlations with socioeconomic factors and caregiver characteristics were of the expected sign, although magnitudes were mostly low or very low. Also, as expected, scores were higher for centers with child-caregiver ratios below the median, in urban areas, where families pay at least $5 for subscription and where the caregiver received training, and lower where the caregiver was indigenous. However, the differences in means were not always statistically significant between the groups and varied by instrument.

Concurrent validity results were strongest when like was compared with like, such as the ITERS-R and the CC-IT-HOME, the two instruments with a combination of structural and process quality items. On the other hand the CLASS had low correlations with all three other instruments. This could be explained by the fact that these instruments are measuring different (and potentially complementary) quality aspects i.e., CLASS is more process quality focused. Correlations between ITERS-R and CLASS in Ecuador were lower than those in the validation study of CLASS in the U.S. [19] e.g., correlations between Space and Furnishings of the ITERS-R and CLASS dimensions in our sample are one third to half of those in the U.S. These lower correlations might be explained by the overall low variability in the quality scores in Ecuador, which would limit the magnitude of common variance with the CLASS. However, other scales such as Personal Care Routines of the ITERS-R showed more comparable results to the U.S. with Positive Climate (*r* = 0.35 in Ecuador vs. *r* = 0.38 in the U.S.), Teacher Sensitivity (*r* = 0.35 vs. *r* = 0.48), and Behavior Guidance (*r* = 0.33 vs. *r* = 0.35).

### Limitations

The study had a number of limitations. First the analysis was conducted in a specific context of low quality. Even though we were able to exploit the variation in scores within this low quality range, it is possible that the overall low variability in quality “masked” some of the correlations or factor loadings in our analysis. The results reported here may differ if the study were replicated in a context of higher quality than that of the centers in our sample. We therefore recommend replicating this study in other contexts to ensure the external validity of the results obtained here.

Because we only chose one classroom per center, another limitation is that we were unable to run a center fixed effects strategy. The advantage of such a strategy would have been to control for all time-invariant characteristics of centers and the population that they serve (for example, differences in socioeconomic status across neighborhoods). The lack of child development outcomes as construct validators for our instruments was also an important limitation. Although this issue was partially addressed by validating the instruments against proxies for family socioeconomic status and characteristics of the center and caregivers, we strongly recommend replicating this exercise while also administering a measure of child development.

Finally, three measures were scored by field observers, while CLASS was scored in a laboratory using filmed classroom observations. Although this latter method increased the quality of CLASS data, it also reduced the comparability of the scores across the four measures and may explain, in part, the low correlation of the CLASS with the other measures. Despite these limitations, the instruments’ administration protocols in the field were highly rigorous, generating a high-quality dataset, which gives us confidence in the internal validity of the results of this analysis.

### Implications for policy and future work

The LAC region is undergoing a paradigm shift from an emphasis on keeping children safe while the caregivers are at work to one in which child care centers foster child development. As a consequence, there is a growing interest from government ministries in LAC to monitor process quality, and particularly child-caregiver interactions, for overall program quality consolidation. The validation exercise in this paper supports the administration of one or more of these instruments in Ecuador (or other similar contexts in the region) and provides useful information for policy-makers and researchers interested in using these instruments for quality monitoring or research purposes.

A strength common to all four instruments is the inclusion of direct observation of caregiver-child interactions. Observation is advantageous in that it can produce data with a higher level of objectivity than that obtained from the alternative caregiver report, which may be subject to self-reporting bias. In addition, empirical evidence shows that direct observation used to evaluate quality predicts child outcomes to a significantly greater degree than interviews or checklists [17]. However, direct observation can be time consuming, as the observer may need to wait until a particular action occurs. In addition, coding of observed sessions can be complex and generally requires more training than for an interview. The CLASS is an extreme example, requiring two weeks of formal training and practice. However, the ITERS-R and MITRCC were also complex and difficult to code, reflected in long administration times (3.5 hours) for both and low IRR (56%) for the MITRCC.

In terms of constructs, the CLASS was the only instrument that focuses exclusively on a broad range of process quality elements. Unfortunately, the video-recording and off-site coding requirements, as well as the ethical considerations of filming children, make the CLASS impractical and too costly for routine monitoring of daycares in Ecuador or other low-resource settings. However, less frequent (e.g., every 2–5 years) center evaluations with the CLASS would make its administration more affordable, as has been demonstrated in the U.S. with Head Start, and would provide valuable information for future policy and program reform in Ecuador.

However, this still leaves unanswered the question of how to conduct *frequent* monitoring in low-resource settings. Frequent administration of the other three instruments could also remain a challenge due to the difficulty of ensuring adequate observer profiles and the on-going intensive training required for the proper administration of a complex instrument over time. For this reason, most policy-makers in the region are looking for ways to incorporate simple, affordable, and culturally-appropriate monitoring tools in routine assessments of process quality in child care centers. Moreover, there remains an urgent need in the region to monitor structural quality, including facilities, safety, materials, and classroom organization. Therefore, the inclusion of both structural and process quality constructs is an advantage of the non-CLASS instruments in that the two aspects of quality are likely to be synergistic with dynamic complementarities over time. For example, having access to a variety of engaging learning materials can provide more opportunities for caregivers to interact meaningfully with children.

Of the four instruments, the CC-IT-HOME was both the easiest to code (with excellent IRR) and the shortest to administer (~ 1hr), making the instrument the most feasible choice for frequent monitoring at scale in a low-resource context such as Ecuador. However, some of the domains of the CC-IT-HOME had low internal consistency (e.g., Organization) in our study, suggesting that a detailed investigation into the content, cultural adaptation and measurement properties of those domains is needed going forward. In addition, the CC-IT-HOME could be enhanced with new modules that capture domains of process quality that are currently not included, but that are valued in the local context based on discussions with local experts. To this end, in our future work, we plan to explore how well the subscales and items from each of the instruments captured different aspects of process quality in order to inform the selection of culturally-appropriate and cost-effective process quality indicators for frequent administration at scale in child care centers in Ecuador, as well as other Latin American countries.
